# A dynamic self-regulation actuator combined double network gel with gradient structure driven by chemical oscillating reaction†

**DOI:** 10.1039/c9ra02340b

**Published:** 2019-04-30

**Authors:** Jie Li, Xiuchen Li, Zhaohui Zheng, Xiaobin Ding

**Affiliations:** Chengdu Organic Chemicals Co. Ltd., Chinese Academy of Sciences Chengdu 610041 China xbding@cioc.ac.cn zhzheng@cioc.ac.cn +86 028 85233426 +86 028 85233426; University of Chinese Academy of Sciences Beijing 100081 China

## Abstract

Self-regulation of the dynamic actuation of a chemical oscillating reaction-based gel was realized by altering the network structure of the gradient double network gel. We demonstrated that the propagation mode of the chemical wave was influenced by the network structure, and consequently determined the dynamic feature of the gel actuator.

## Abstract 2

Self-regulation of the dynamic actuation of a chemical oscillating reaction-based gel was realized by altering the network structure of the gradient double network gel.

## Graphical abstract

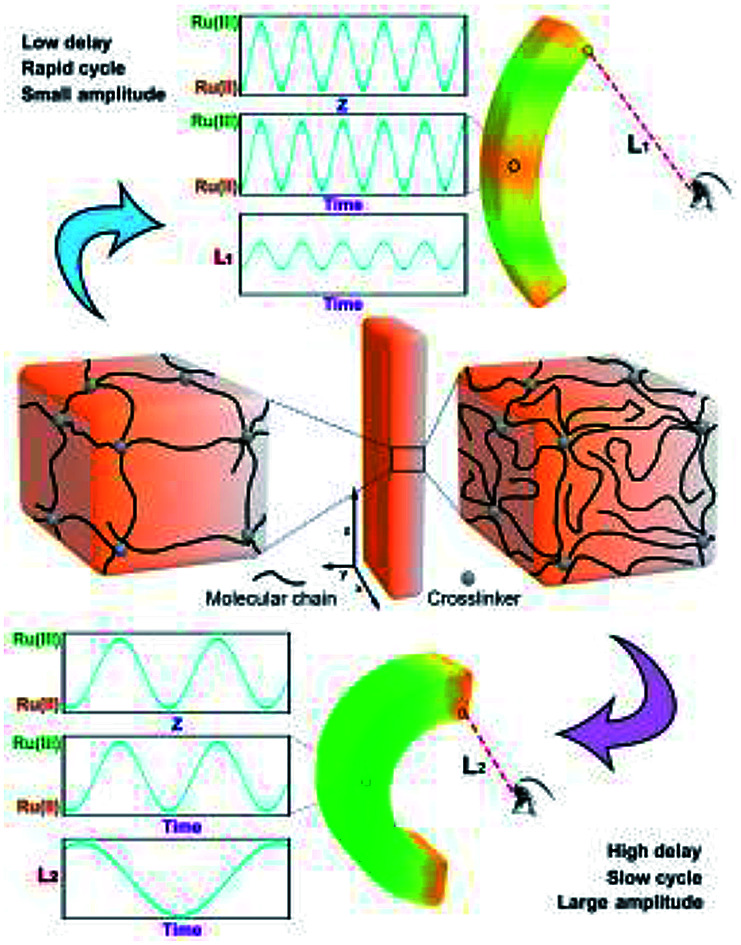

Soft actuators, based on the adaptive deformation of stimuli responsive materials, have the potential to significantly improve healthcare, manufacturing and robotics.^1^ Compared with their rigid counterparts, soft actuators are easily responsive to environmental variations, resilient to high loads, and flexible enough to be compliant and safe to humans.^2^ Especially on the nanometer to centimeter scale, soft actuators become a strong promoting force for the development of artificial muscles, microsensors, microrobots, *etc.*^3^

In the last two decades, a type of “self-oscillating gel” based on chemical oscillating reaction (COR) has attracted a lot of attention due to its similarity to natural rhythmic phenomena (*e.g.* heartbeat, brain waves, the *Turnera ulmifolia* Linn. flower). Differing from those ON–OFF switching-dependent actuators, the COR-based gel actuators showed self-sustained actuation. The continuous cyclic actuation of this gel can continue for several minutes in a closed system.^4^ Furthermore, by altering the geometric shape and size of the self-oscillating gel, a series of interesting biomimetic functions have been developed, including looper motion, ciliary motion, cellular beating, *etc.*^5^

Up to now, the diversification of deformation mode of the self-oscillating gel has been achieved by changing the shape of the gel. However, the dynamic feature of existing systems, such as actuation frequency and amplitude, is uncontrollable in the consistent environmental condition. In the potential application fields (*e.g.* microsensors and robots), the precise control of gel actuation is significant, and existing systems can not meet the demand. Therefore, regulation of the dynamic features (*e.g.* amplitude, period and delay) of COR-based gel actuator remains a great challenge in the field.

To solve this problem, altering the COR parameters^6^ (*e.g.* concentration of substrates, temperature) or the material structure are alternative ways. However, the COR parameters must be restricted within narrow limits to ensure the synchronism of responsive actuation and COR variation, otherwise the stimuli responsive behavior cannot be realized. Therefore, altering the material structure to regulate the dynamic actuation is the rational option.

In the reported COR-based gel systems, the directional propagating chemical wave in the gel plays an important role in realizing the function. For instance, the mass transportation speed of the gel strip is directly determined by the chemical wave velocity.^7^ As is known, the propagation mode of wave (*e.g.* light wave, sound wave and electric wave) is related to the properties of the propagation medium, and the parameters of the wave (wavelength and velocity) will change when the wave propagates in different medium. In the case of COR-based gel systems, the COR substrate-swelled gel network can be regarded as the propagation medium of chemical wave. It is easy to imagine that if the propagation mode of chemical wave can be tuned by altering network structure, the dynamic features of the gel will be regulated at the same time.

Based on the above consideration, we aim at regulating the dynamic actuation of the gel by altering network structure. Since the COR-responsive gel is a crosslinked network of copolymers, the proper monomer ratio is crucial in achieving responsive actuation. Altering the network structure (monomer content or crosslinking density) may change the proper monomer ratio and lead to the failure of responsive actuation.

The interpenetrating double network gel is able to combine the functions of both networks without changing their respective monomer ratio, such as those double network gels possessing multi-responsiveness.^8^ Inspired by this, we designed an interpenetrating double network gel with tunable primary network and COR-responsive secondary network. The structure of the primary network is varied to tune the chemical wave. The monomer ratio of the secondary network is consistent to ensure the responsive actuation. Based on the varied propagation mode of chemical wave in different media, the dynamic features (characterized by motion delay, motion period, motion amplitude) of the gel can be regulated by altering the primary network structure (monomer content or crosslinking density). By utilizing the method developed in our previous work,^9^ the secondary network is designed to be asymmetric to carry out bending-stretching actuation. By utilizing COR as the *in situ* autonomous driving source with periodic variation, the gel undergoes continuous and cyclic bending-stretching actuation with diversified actuation mode in closed COR substrate solution (Scheme 1).

**Scheme 1:**
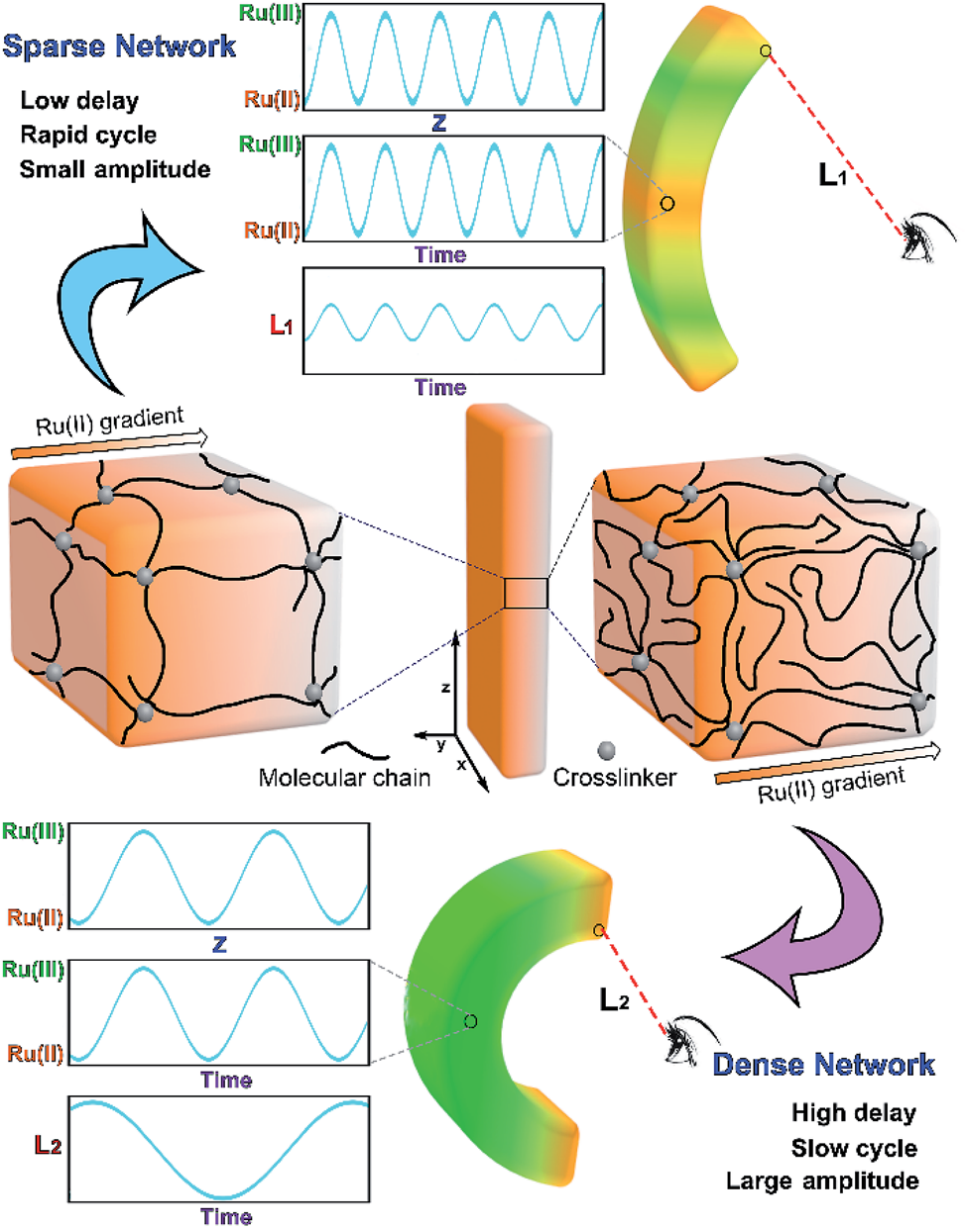
Conceptual illustration of self-regulation of the dynamic actuation of chemical oscillating reaction-based gel by altering network structure.

In this study, we prepared a series of mobile gels (abbreviated as MGs) *via* a two-step method. For each MG, a polyisopropylacrylamide (PNIPAAm) gel was synthesized first *via* free radical polymerization, and vinylated micro-gel (VMG) was used as the cross-linker to ensure the motility of the MGs.^10^ Second, asymmetric poly(Ru(ii)(bpy)_3_-*co*-NIPAAm) network was fabricated by rapidly fixing the spontaneous concentration gradient of the gelating solution during natural infiltration process *via* photopolymerization.^11^ In this way, an interpenetrating PNIPAAm/poly(Ru(ii)(bpy)_3_-*co*-NIPAAm) double network gel was prepared (Fig. 1). Detailed preparation method can be seen in ESI.† By utilizing the same method, a series of MGs with varied PNIPAAm network structure (diverse monomer content and crosslinking density) were prepared, and the structural profiles of the MGs are shown in Table 1. The MGs were arranged in the increasing order of monomer content (MG 1 < MG 2 < MG 3) and crosslinking density (MG 4 < MG 5 < MG 6). Belousov–Zhabotinsky (B–Z) reaction (a typical COR) was selected as the *in situ* driving source to induce the continuous cyclic bending-stretching actuation of the prepared MGs. The B–Z reaction substrates were aqueous solution of [HNO_3_] = 0.89 M, [NaBrO_3_] = 84 mM, [malonic acid] = 62.5 mM. B–Z reaction can be initialized in the presence of the catalyst Ru(ii)(bpy)_3_ which is covalent bonded in the gel.

**Fig. 1:**
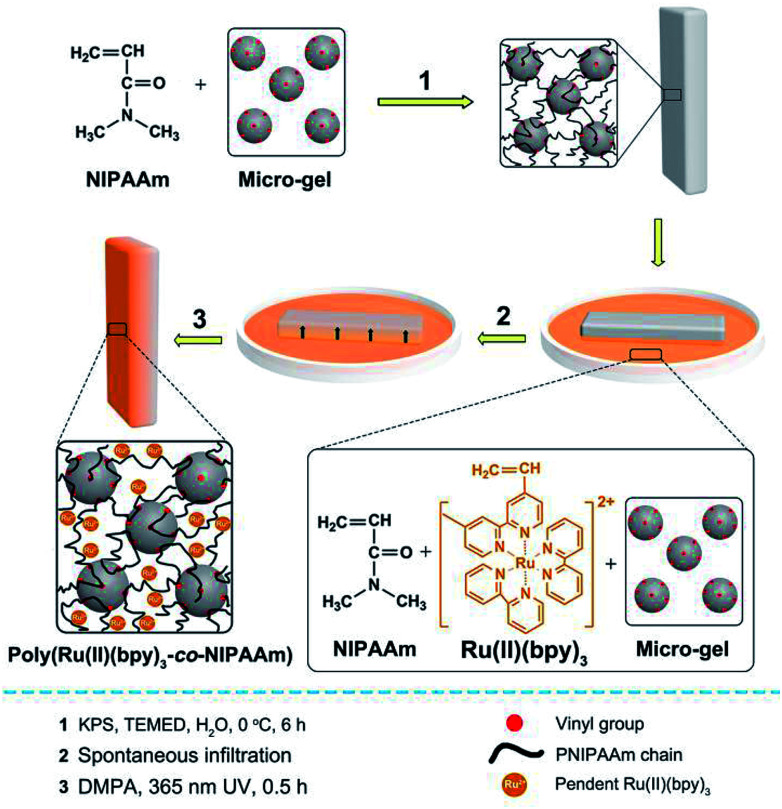
Preparation method of the interpenetrating PNIPAAm/poly(Ru(ii)(bpy)_3_-*co*-NIPAAm) double network gel (MG) with asymmetric distribution of Ru(ii)(bpy)_3_.

**Table 1:** Structural profiles of the MGs

	NIPAAm^a^ (mol L^−1^)	Micro-gel^a^ (×10^−4^ g mL^−1^)	Poly(Ru(bpy)_3_-*co*-NIPAAm) network^b^ (*m*%)	Monomer content^c^ (*m*%)
MG 1	0.5	23	14.4	7.2
MG 2	1	23	14.4	14.3
MG 3	2	23	14.5	30.1
MG 4	1	11.5	14.4	14.1
MG 5	1	23	14.3	14.3
MG 6	1	46	14.3	14.9

a In gelating solution of PNIPAAm network.

b Determined by weighing the gel in thoroughly dry state before and after the construction of poly(Ru(ii)(bpy)_3_-*co*-NIPAAm) network.

c Determined by weighing the gel in thoroughly dry state and equilibrium swelling state.

By immersing the MGs in B–Z reaction substrate solution, B–Z reaction occurred in all the MGs. At first, the MGs underwent an induction period, because the B–Z substrate needs to permeate into the gels and reach equilibrium. After that, B–Z reaction was initiated by the catalyst Ru(ii)(bpy)_3_ in the gels. Due to the temporal-spatial character of B–Z reaction,^12^ the gel cannot be synchronously oxidized or reduced. A chemical wave of redox states was generated and propagated in the gel periodically. Driven by the B–Z reaction, the swelling ratio of the gels (MG 1, MG 2 and MG 3) varied according to the periodic change of Ru(ii)(bpy)_3_ redox state (Ru(ii)–Ru(iii)). Due to the asymmetric distribution of Ru(ii)(bpy)_3_, the periodic swelling-deswelling variation of the gel was asymmetric, resulting in cyclic bending-stretching actuation of the gel, as shown in Videos S1–S3 ESI.† For the MGs with increasing cross-linking density (MG 4, MG 5, MG 6), only MG 5 could accomplish the bending-stretching actuation. We believe that the motility of MGs heavily depends on cross-linking density. In the case of deficient cross-linking density, the conformational change of molecular chain is insufficient to cause the macroscopic actuation of the gel due to the relatively longer molecular chain. On the contrary, the excessive cross-linking density confines the conformational change of molecular chain and the gel becomes too rigid to accomplish the bending-stretching actuation.

The propagation mode of chemical wave is diverse in MGs (MG 1, MG 2 and MG 3), as shown in Fig. 2a. Since the initial position of B–Z reaction in aqueous phase is random,^13^ the chemical wave may propagate inside the gel from two ends to the center (MG 1), from center to both ends (MG 2), or from one end to the other (MG 3). As is mentioned above, the B–Z substrate-swelled MGs can be regarded as the media in which the chemical wave propagates. In the gel with lower monomer content, there is a large quantity of aqueous solution but a small quantity of polymer chains (see Table 1). The gel is a sparse medium for chemical wave, thus the chemical wave propagates with short wavelength at high velocity. Oppositely, the chemical wave propagates with long wavelength at low velocity in the gel with higher monomer content, which is a dense medium for chemical wave, as shown in Fig. 2b.

**Fig. 2:**
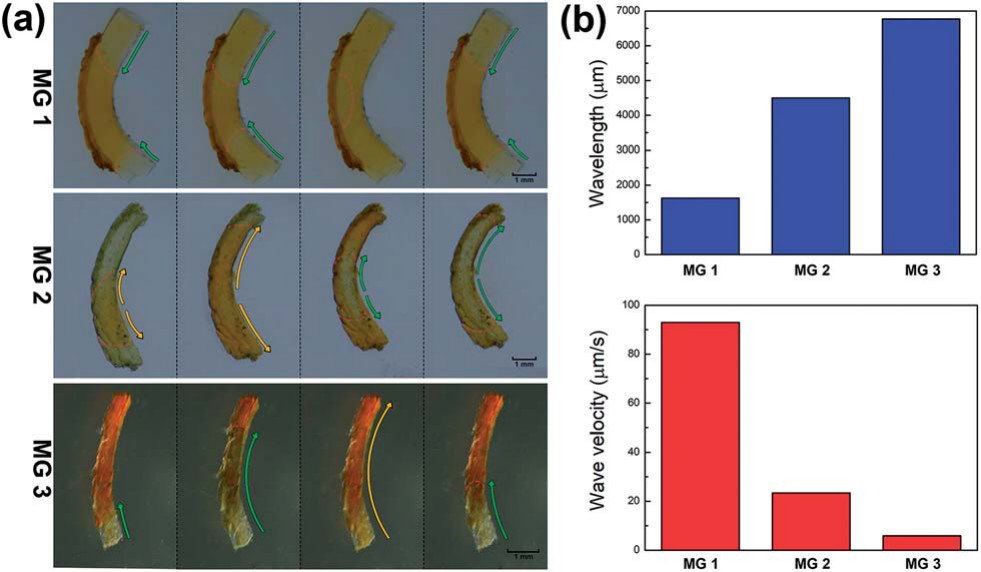
(a) Image of chemical wave of redox variation propagating in the MGs. Red dash lines show the boundary of oxidized region and reduced region. Coloured arrows indicate the direction and range of chemical wave, green arrows represent the wave of Ru(iii) state and orange arrows represent the wave of Ru(ii) state. (b) Parameters of the chemical waves propagating in MGs. Both wavelength and wave velocity are the average value of observed B–Z reaction processes.

Since the chemical wave is the *in situ* driving source to induce the responsive actuation of MGs, varied propagation mode of chemical wave leads to diverse dynamic actuation of MGs. To characterize the various cyclic actuation of MGs, an assistant point (defined as O) was set in permanent position in the observed area. The minimum distance between O and selected position A on the MGs was defined as *L*, the displacement of *L* over time was expressed as Δ*L* (Fig. 3a). For the MGs with increasing monomer content (MG 1, MG 2, MG 3), the bending-stretching actuation was carried out in different modes, as shown in Fig. 3b. To clarify the relationship between the chemical wave propagation mode and the dynamic actuation of the gel, the continuous cyclic bending-stretching actuation of MGs was quantitatively analyzed in induction period, motion period and motion amplitude, respectively.

**Fig. 3:**
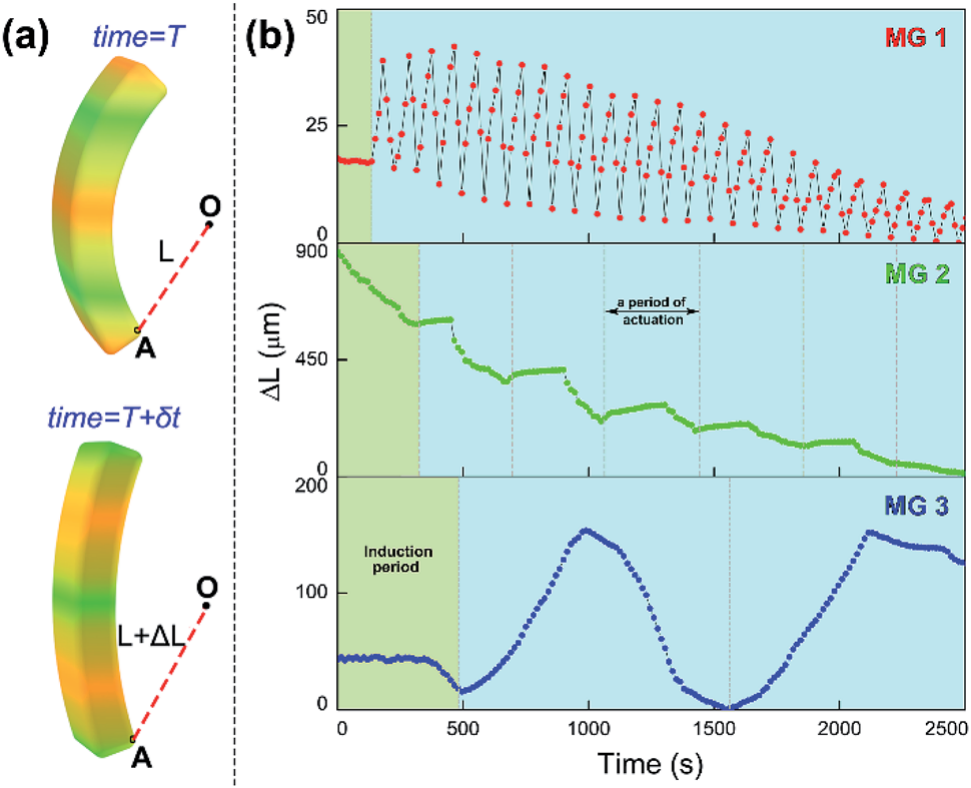
Continuous cyclic actuation profiles of MGs in the solution of BZ substrates ([HNO_3_] = 0.89 M, [NaBrO_3_] = 84 mM, [MA] = 62.5 mM) at 20 °C. (a) Illustration of quantitative method. (b) Actuation profiles of MGs, the time interval of data collection is 15 s.

It can be seen in Fig. 4 that the time duration of induction period is negatively correlated with monomer content of the gel, because the gel with lower monomer content possesses higher infiltration rate of B–Z substrate and needs less time to initiate the B–Z reaction, and *vice versa*. The motion period of MGs is positively correlated with monomer content. As is mentioned above, the MG with lower monomer content possesses short wavelength and high velocity, thus the frequency of alternating driving source for the MG is higher (*f* = *ν*/*λ*), and *vice versa*. Due to the micro-gel cross-linking,^10^ the MGs are rapidly responsive to the variation of driving source. As a result, higher frequency of driving source (*i.e.* redox states variation in B–Z reaction) leads to shorter motion period of the gel, and *vice versa*. The motion amplitude of MGs is determined by the alternating character of B–Z reaction and the stress–strain character of polymer material. It is well known that there is “dynamic mechanical loss” when polymer material is under alternating force. Similarly, the cyclic actuation of MGs is caused by the stress action of asymmetric swelling-deswelling behavior of polymer network, which is induced by an alternating driving source (B–Z reaction). For the MG with lower monomer content, higher alternating frequency of chemical wave leads to serious dynamic mechanical loss, resulting in lower motion amplitude, and *vice versa*. Thus, the motion amplitude of MGs is positively correlated with monomer content. Besides, it can be seen in Fig. 3b that there is obvious attenuation of motion amplitude for MG 1 and MG 2. This is caused by the reduction of concentration of malonic acid in the system. According to the mechanism of B–Z reaction, malonic acid can be regarded as the fuel to support the continuous actuation of the gel^13^ and the dissipation of malonic acid results in the attenuation of motion amplitude.

**Fig. 4:**
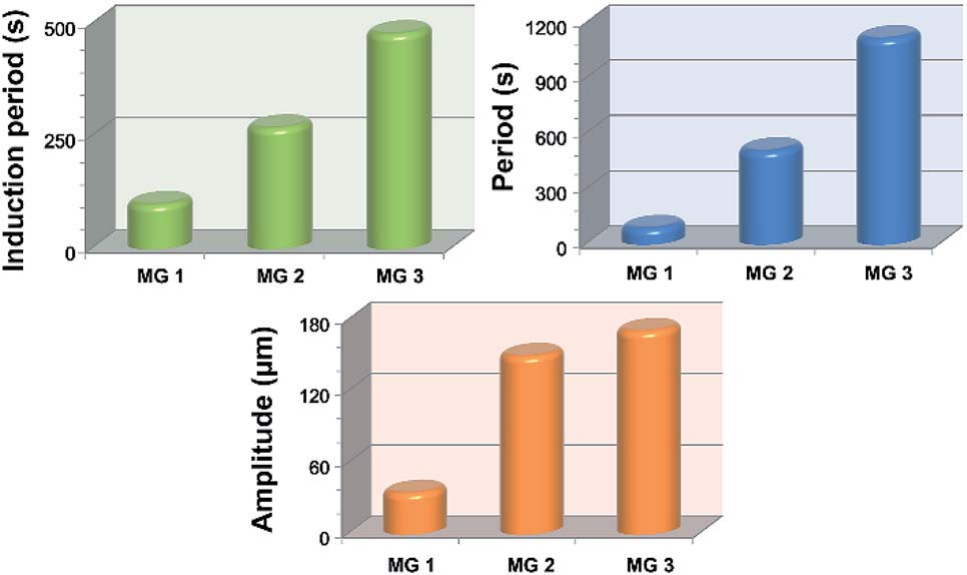
Motor parameters of the MGs. The introduction period is the time period from the immersion of the gel to the beginning of bending-stretching process. The motion period and motion amplitude for MG 1 and MG 2 are average value of all observed cycles.

To summarize, the frequency of chemical wave is determined by the density of MG. The MG undergoes rapid actuation with small amplitude when the frequency of chemical wave is higher and undergoes slow actuation with large amplitude when the frequency is lower.

It is worth mentioning that researchers have simulated a series of precisely controlled directional movement of gels driven by B–Z reaction (*e.g.* gel train and gel pinwheel) by utilizing computational modeling.^14^ In the mathematical model, the dynamic feature of the gel is totally controlled by the manual set diffusion factor and concentration factor. In our work, we found the monomer content is one of the forms of the abstract diffusion factor. Computational modeling works and ours are mutually supportive and jointly reveal the rule of motion of the gel system with continuous cyclic actuation based on COR.

In conclusion, we have realized for the first time the self-regulation of dynamic actuation of COR-based gel actuator by altering the network structure of gradient double network gel. The results indicate that dynamic feature of the double network gel is correlated with the propagation mode of chemical wave which is determined by the network structure. This work provides an opportunity to understand the law of motion of soft actuators driven by an alternating stimuli. We believe that novel soft actuators with precisely controlled dynamic actuation will be developed in the light of the rules this work revealed.

## Conflicts of interest

There are no conflicts to declare.

## Supplementary Material

RA-009-C9RA02340B-s001

RA-009-C9RA02340B-s002

RA-009-C9RA02340B-s003

RA-009-C9RA02340B-s004
