# Supplementary material for: A dynamic self-regulation actuator combined double network gel with gradient structure driven by chemical oscillating reaction
Source: RSC Adv. 2019 Apr 30;9(23):13168–72. doi: 10.1039/c9ra02340b (PMC9063756; doi:10.1039/c9ra02340b)
Supplement: RA-009-C9RA02340B-s001 [file RA-009-C9RA02340B-s001.pdf]

Electronic Supplementary Material (ESI) for RSC Advances.
This journal is © The Royal Society of Chemistry 2019

Electronic Supporting Information

Dynamic self-regulation actuator combined double network gel with gradient structure driven by chemical oscillating reaction

Jie Li,[a,b] Xiuchen Li,[a,b] Zhaohui Zheng,[*a] and Xiaobin Ding[*a]

[a]*Chengdu Organic Chemicals Co. Ltd., Chinese Academy of Science, Chengdu 610041, China.*

[b]*University of the Chinese Academy of Science (CAS), Beijing 100049, China*

E-mail: xbding@cioc.ac.cn; zhzheng@cioc.ac.cn; Fax: +86 28 85233426; Tel: +86 28 85233426

Materials and Methods

Ruthenium (III) chloride hydrate ($RuCl_3·3H_2O$, 99 %), 4,4’-dimethyl-2,2’-dipyridyl, ammonium hexafluorophosphate ($NH_4[PF_6]$), N-butyllithium (2.2 mol/L in hexane), sodium bromate ($NaBrO_3$), malonic acid (MA), N-isopropylacrylamide (NIPAAm), N,N’,N’,N’-tetramethylethylenediamine (TEMED), xylene were purchased from Aladdin Reagent (Shanghai, China). 2,2’-bipyridine, lithium chloride (LiCl), sodium dodecyl sulfate (SDS), methacrylic acid (MAA), diisopropylamine, dimethyl formamide (DMF), tetrahydrofuran (THF) were purchased from TCI reagent Co., Ltd (Shanghai, China). Cerium (III) sulfate and cerium (IV) sulfate were purchased from Sinopharm Chemical reagent Co., Ltd (Shanghai, China). Sodium bicarbonate ($NaHCO_3$), potassium persulfate (KPS), phosphorus pentoxide (P_2O_5), ether, acetone, dichloromethane (DCM), paraformaldehyde, nitric acid (HNO_3, 68 %), methanol, dimethyl sulfoxide (DMSO) were purchased from Kelong Chemicals (Chengdu, China). N,N'-methylenebisacrylamide (MBA), 2,2-dimethoxy-2-phenylacetophenone (DMPA), were purchased from J&K Chemicals (Beijing, China) and recrystallized before use. The deionized water used in this study was lab made.

Characterizations

^1H NMR spectra were recorded on a 300MHz Bruker Avance-300 NMR spectrometer, and chemical shifts were reported in parts per million (ppm) in reference to the residual protons of the deuterated solvents. FTIR spectra were obtained on a FTIR spectrophotometer (Nicolet MX-1E) using KBr pellet pressing method. Particle size analyze was performed on a Malvern laser particle size analyzer (mastersizer 2000), and the dispersing agent was water. On-line observations of the gel system were carried out on an Olympus stereomicroscope with image capture system. Image

analysis was carried out by Olympus Cellsens standard software.

Synthesis of Ru(II)(bpy)$_3$ monomer

The Ru(II)(bpy)$_3$ monomer ruthenium (4-vinyl-4'-methyl-2,2'-bipyridine)-bis-(2,2'-bipyridine) - bis-(hexafluorophosphate) (Ru(vmpy)(bpy)$_2$(PF$_6$)$_2$) was synthesized according to published literature[1,2] with some improvements, The detailed procedure was as follow.

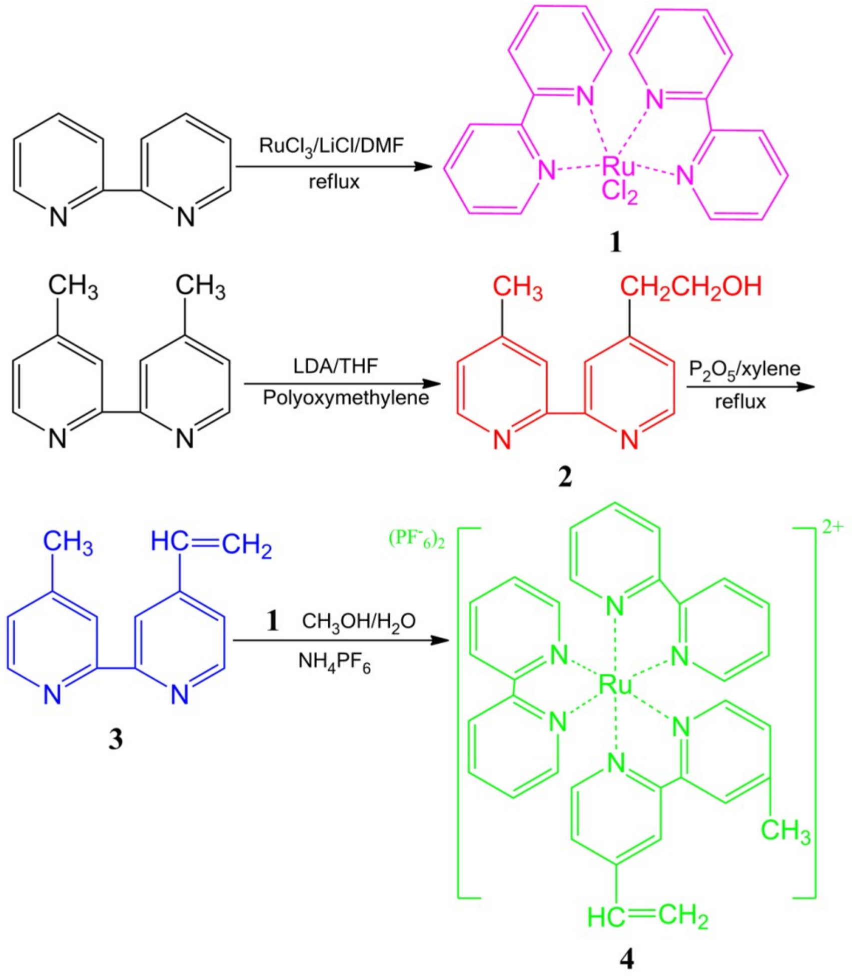

Figure S1 Synthesis route of Ru(vmpy)(bpy)$_2$(PF$_6$)$_2$

Synthesis of 1: 7.8 g $RuCl_3·3H_2O$, 9.36 g 2,2'-bipyridine and 8.4 g LiCl were added to 50 mL DMF, then the mixture was refluxed for 9 h. 250 mL acetone was poured into the mixture and kept overnight. After filtration, the solid was gently washed by deionized water until the filtrate turned to green then washed by ether for three times. Vacuum drying gave a white solid (6.62 g,

yield: 53 %).

Synthesis of 2: 25.6 mL N-butyllithium and 8.0 mL diisopropylamine were added to 30 mL THF in a three-neck flask, the solution was thoroughly stirred. 10 g 4,4'-dimethyl-2,2'-dipyridyl in 250 mL THF was slowly added to the three-neck flask from a dropping funnel, and the color turned to brown. 2 h later, 1.7 g paraformaldehyde was added in, the reaction continued for over 24 h, the mixture gradually turned to green and opaque. The mixture was quenched with ice water and extracted with ether, then evaporated. The crude product was purified via column chromatography (DCM: MeOH=15:1) to get viscous liquid (7.63 g, yield: 65 %). ^{1}H NMR (300MHz, $CDCl_3$): δ 8.55-8.57 (d, 2 H), 8.20-8.25 (d, 2 H), 7.12-7.26 (q, 2 H), 3.93-3.97 (t, 2 H), 2.92-2.96 (t, 2 H), 2.43 (s, 3 H).

Synthesis of 3: 7.5 g of **2** in 200 mL of xylene was reflux with 30 g P_2O_5 for 2 h, then the mixture was cooled and ice cube was added to decompose the excess P_2O_5. The aqueous layer was separated and the organic layer was extracted with DCM. By evaporation of DCM and purification via column chromatography (DCM: MeOH=15:1), a white solid was obtained (4.43g, yield: 60 %). ^{1}H NMR (300MHz, $CDCl_3$): δ 8.61-8.63 (d, 1 H), 8.54-8.56 (d, 1 H), 8.41 (s, 1 H), 8.25 (s, 1 H), 7.30-7.32 (q, 1 H), 7.15-7.16(d, 1 H), 6.72-6.81 (q, 1 H), 6.07-6.13 (d, 1 H), 5.51-5.54 (d, 1 H), 2.45 (s, 3 H).

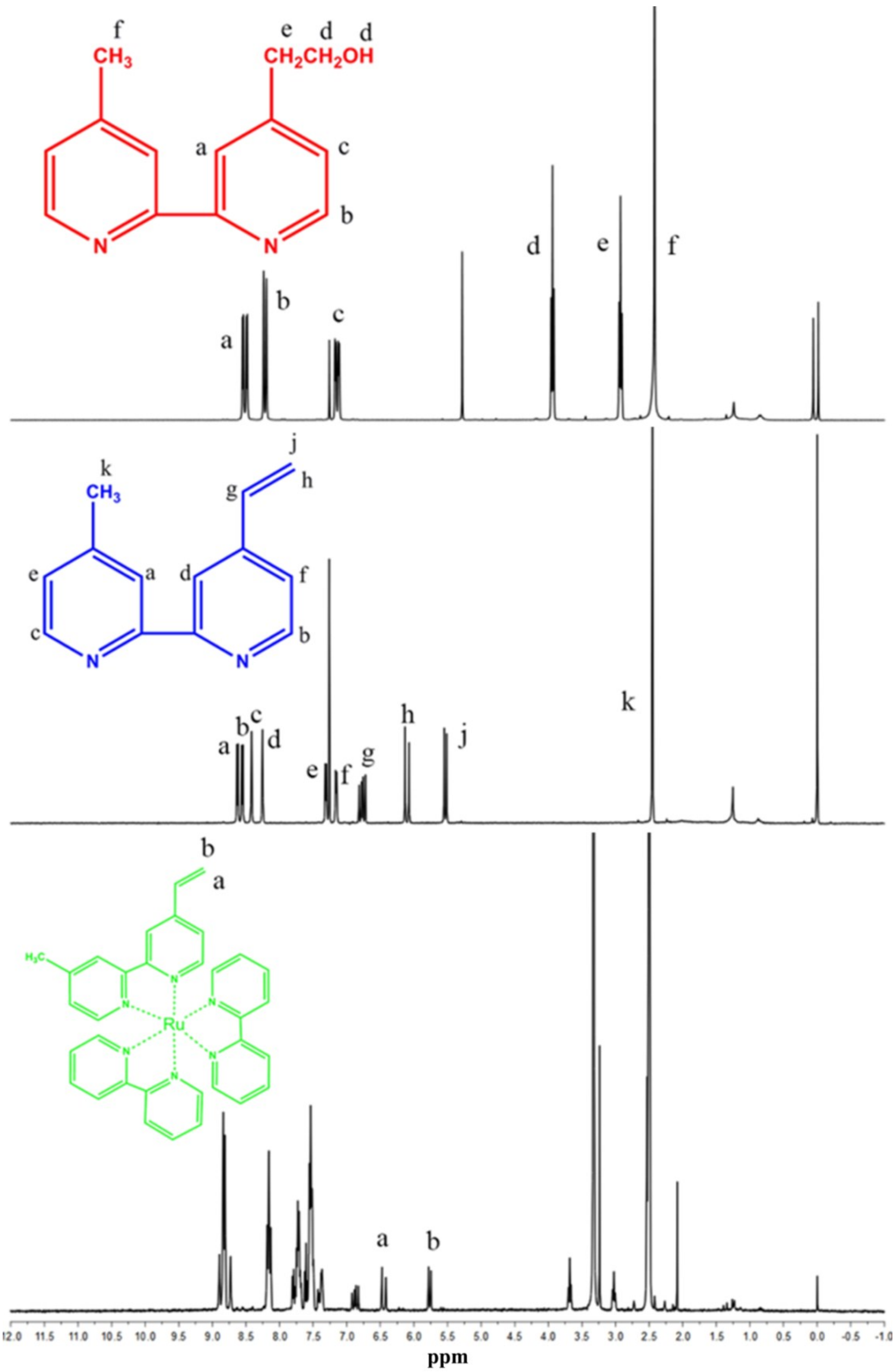

Figure S2 ^{1}H NMR spectra of intermediate products (**2** and **3**) and the target product ($Ru(vmpy)(bpy)_2(PF_6)_2$)

Synthesis of 4: 1 g of **1**, 2.65 g of **3** and 1.52 g $NaHCO_3$ were refluxed together in 60 mL methanol-water (2:3 v/v) solution, until the mixture turned to blood red. 4 mL aqueous $NH_4[PF_6]$

was added and a red solid precipitated out. The product (3.45 g, yield: 70 %) was obtained via recrystallization from acetone/DCM (1:1 v/v) solution. ^1H NMR (300MHz, acetone-d_6): δ 6.82-8.89 (aromatic moiety, 22 H), 6.41-6.47 (d, 1 H), 5.74-5.78(d, 1 H), 2.49 (s, 3 H).

Preparation of the vinylated micro-gel crosslinker

The vinylated micro-gel was prepared via a two-step method, and the preparation process is as follow:

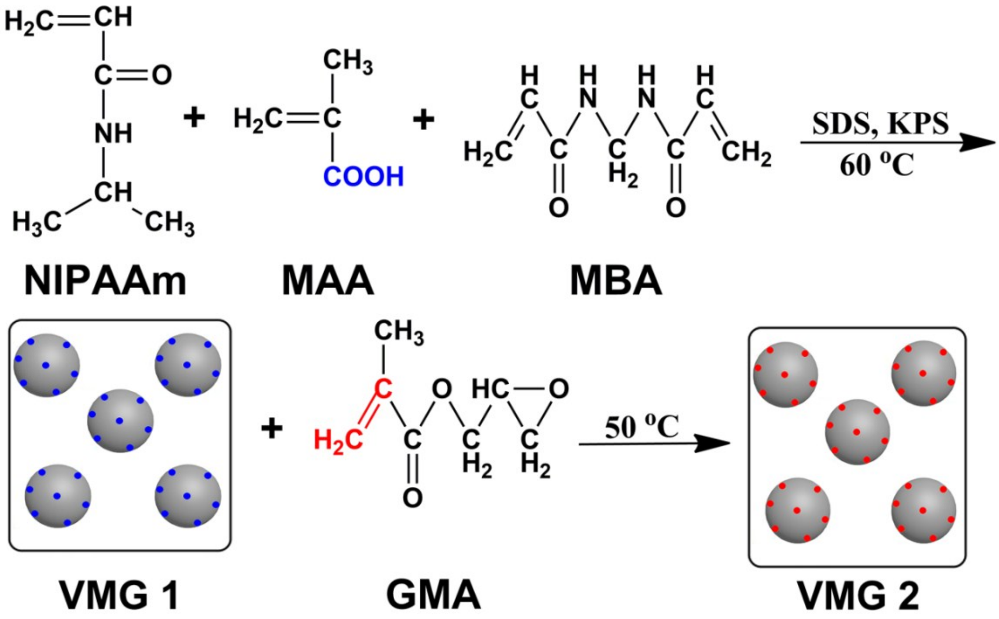

Figure S3 Preparation process of vinylated micro-gel

Synthesis of VMG 1: 50 mL of deionized water was added in a flask and bubbled with N_2 for 5 min. 0.99 g NIPAAm, 0.495 g MAA, 0.015 g MBA and 0.034 g KPS were added in the flask, and then the solution was thoroughly stirred. After that, 0.338 g SDS was added in, and the flask was sealed immediately. The polymerization was carried out for 5 h at 60 °C with mechanical stirring at 300 r.p.m. To remove SDS in the VMG 1 dispersion, the dispersion was dialyzed against deionized water for 1 week with refreshing water every day.

Synthesis of VMG 2: 0.8 g GMA was added to 50 mL VMG 1 dispersion, the mixture was heated

to 50 ℃ and kept for 8 h with mechanical stirring (350 r.p.m). After that, the VMG 2 dispersion was dialyzed against ethanol for 1 week to remove unreacted GMA. Stretching vibration of carbon-carbon double bond was found at 1690 cm^{-1}. The surface area mean particle size of the VMG 2 was 5.205 μm, and volume average particle size was 6.255 μm.

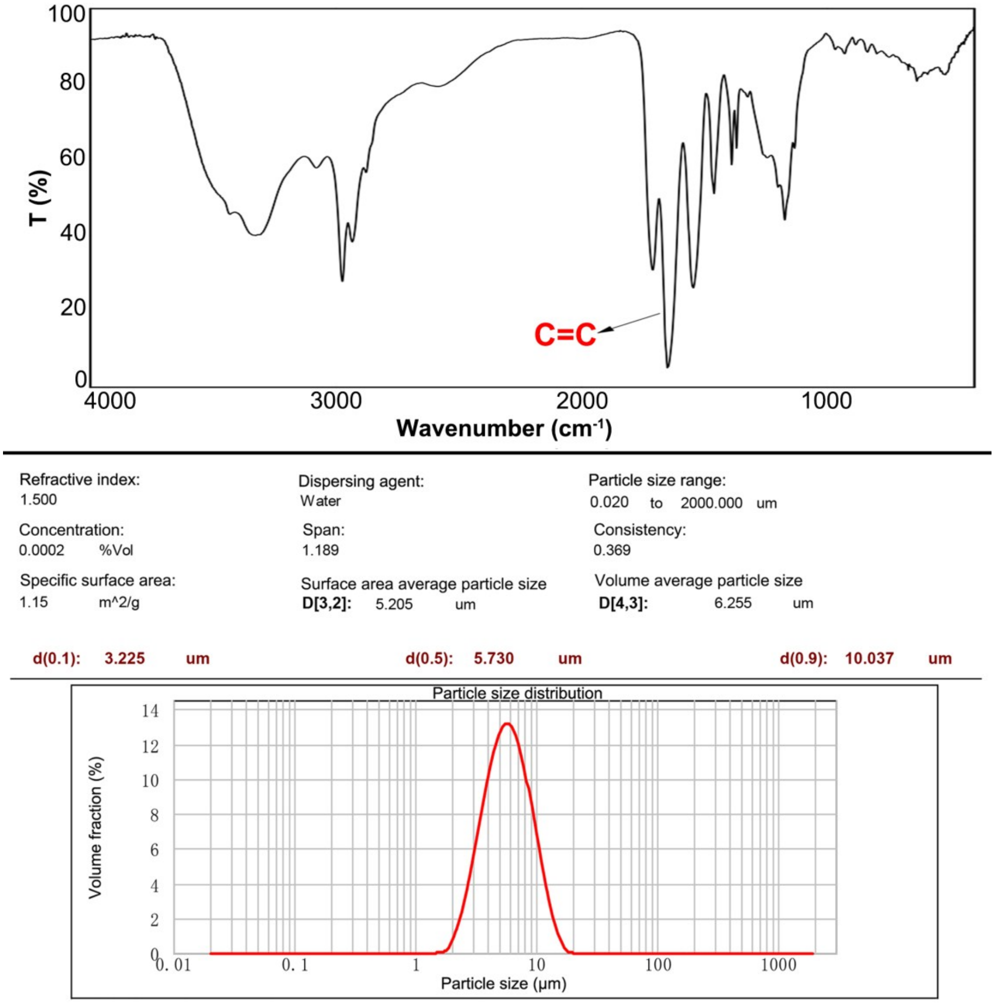

Figure S4 ATR-FTIR spectrum and particle size analysis report of vinylated micro-gel

Solvent exchange of the VMG 2 dispersion: The VMG 2 dispersion was then dialyzed against methanol for 4 days with changing methanol twice a day.

Storage: The obtained VMG 2/water and VMG 2/methanol dispersion was stored at 4 ℃ for subsequent use.

Preparation of the PNIPAAm/Poly(Ru(II)(bpy)$_3$-*co*-NIPAAm) gel

Polymerization of PNIPAAm gel: Quantified NIPAAm and 22.6 mg KPS were dissolved in 10 mL VMG 2/water dispersion (quantified concentration) and the mixture was thoroughly stirred. Then 20 μL TEMED was added to the mixture as polymerization accelerator. The mixture was immediately injected into a self-made mold (two pieces of 10 cm ×10 cm hydrophilic glass separated by a piece of 1 mm thick silicon rubber with a square hole), and the polymerization was carried out at 0 °C for 6 h. The obtained gel was freeze-dried for subsequent use.

Preparation of interpenetrating gel: First, 0.4 g NIPAAm, 0.2 g Ru(II)$(bpy)_3$, 30 mg DMPA and 1.5 mL DMSO were dissolved in 10 mL VMG 2/methanol dispersion, the mixture was stirred for 5 min. Next, the freeze-dried network was partially immerged into the solution for 15 min to form concentration gradient spontaneously. Crosslinking was performed under a 365 nm UV lamp (B100AP, Blak-Ray) for 30 min, a water tank was employed to reduce the heat effect. At last, the prepared gel was subjected to a solvent exchange process by being immersed in a series of graded water-methanol solutions (25, 50, 75 and 100 %) for two days, respectively.

Performance

The continuous cyclic actuation of PNIPAAm/Poly(Ru$(bpy)_3$-*co*-NIPAAm) gels were observed on an Olympus stereomicroscope with image capture system and thermostatic controller. The gels were placed in a 24 well-plate, 2 mL of solution 1 ([HNO_3]=0.89 M, [$NaBrO_3$]=0.168 M) was added in, the gel totally turned to light green after a certain period of time. Then 2 mL of solution 2 ([HNO_3]=0.89 M, [MA]=0.125 M) was added, after the induction period, BZ reaction was initiated in the gel. The entire process of the gel was recorded by image capture system. The image analysis was carried on with Cellsens standard software.

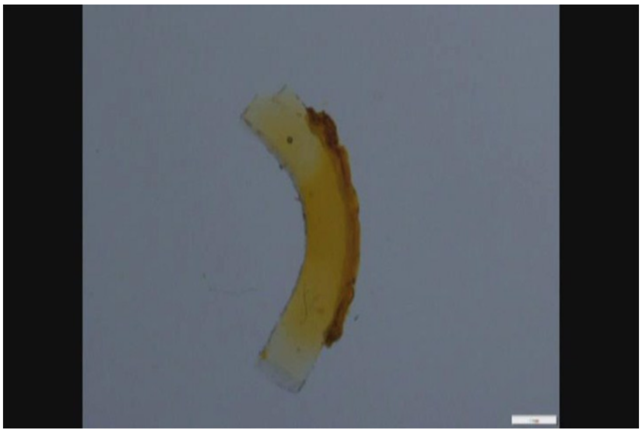

Video S1 shows the continuous and cyclic bending-stretching actuation of MG 1 within 2500 s. Playback speed is 125×.

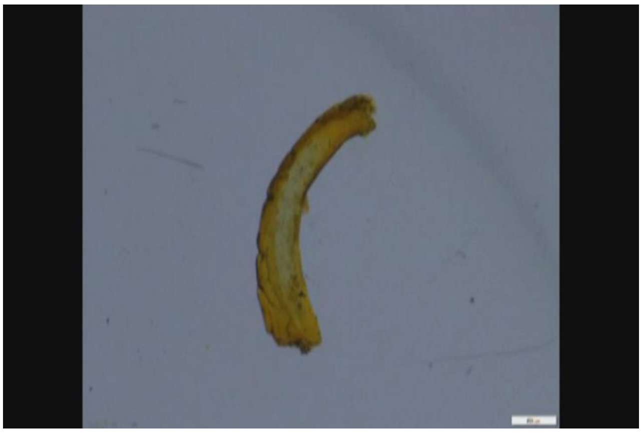

Video S2 shows the continuous and cyclic bending-stretching actuation of MG 2 within 2500 s. Playback speed is 125×.

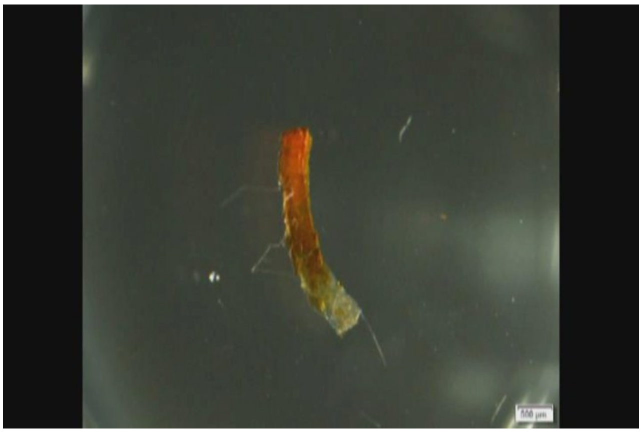

Video S3 shows the continuous and cyclic bending-stretching actuation of MG 3 within 2500 s. Playback speed is 125×.

References

[1] G. Sprintschnik, H. W. Sprintschnik, P. P. Kirsch and D. G. Whitten, *J. Am. Chem. Soc.*, 1977, **99**, 4947-4953.

[2] P. K. Ghosh and T. G. Spiro, *J. Am. Chem. Soc.*, 1980, **102**, 5543-5549.
